# Supplementary material for: ProteinShader: illustrative rendering of macromolecules
Source: BMC Struct Biol. 2009 Mar 30;9:19. doi: 10.1186/1472-6807-9-19 (PMC2672931; doi:10.1186/1472-6807-9-19)
Supplement: Additional file 1 — ProteinShader program without source code. This compressed file contains the complete ProteinShader program including associated libraries, but no source code. A README.txt file gives an overview of the ProteinShader distribution, and the index.html file in the help subdirectory has directions on getting started with the program as well as a set of tutorials. [file 1472-6807-9-19-S1.zip › ProteinShader-beta-0_9_4-binary/help/api/org/proteinshader/structure/Description.html]

Description (ProteinShader API)


|  |  |  |
| --- | --- | --- |
| **Overview** \| **Package** \| **Class** \| **Use** \| **Tree** \| **Deprecated** \| **Index** \| **Help** | |  |
| **PREV CLASS**   **NEXT CLASS** | **FRAMES**    **NO FRAMES**     **All Classes** |  |
| SUMMARY: NESTED \| FIELD \| CONSTR \| METHOD | DETAIL: FIELD \| CONSTR \| METHOD |  |


---


## org.proteinshader.structure Class Description

```
java.lang.Object
  org.proteinshader.structure.Description
```

---

``` public class Description extends Object ```

A Description object serves as a container for the Category objects
that hold Records describing a PDB structure entry.

---

**Constructor Summary**

`Description(String structureID)`
  
          Constructs a Description with the structureID specified as an
argument.


| **Method Summary** | |
| --- | --- |
| `Category` | `addNewCategory(String categoryName)`             Creates a Category, adds it to a hash, and then returns the new Category. |
| `void` | `addNewLine(String categoryName, String recordName, String line)`             Adds a line to the requested Category/Record. |
| `Category` | `getCategory(String categoryName)`             Retrieves a Category by its name. |
| `Record` | `getRecord(String categoryName, String recordName)`             Retrieves a Record by its Category name and Record name. |
| `String` | `getStructureID()`             Returns the ID of the Structure that owns this Description. |
| `Iterator<Category>` | `iteratorCategories()`             Returns an Iterator for the Category objects held by this Description. |
| `int` | `numberOfCategories()`             Returns the number of Category objects held by this Description. |
| `void` | `removeCategory(String categoryName)`             Removes a Category. |

**Methods inherited from class java.lang.Object**

`clone, equals, finalize, getClass, hashCode, notify, notifyAll, toString, wait, wait, wait`

**Constructor Detail**

### Description

```
public Description(String structureID)
```

:   Constructs a Description with the structureID specified as an
    argument.
    The structureID should be for the Structure that owns this
    Description. The Structure that creates this Description should
    have already checked that the structureID has at least one
    character, so it is not checked again here.

    **Parameters:**: `structureID` - the PDB ID of the Structure the Description belongs to.


**Method Detail**

### getStructureID

```
public String getStructureID()
```

:   Returns the ID of the Structure that owns this Description.
    The String returned cannot be null or empty, because the
    constructor checks that this read-only attribute has at least
    one character.

    :   **Returns:**: The PDB ID of the Structure.

---


### addNewLine

```
public void addNewLine(String categoryName,
                       String recordName,
                       String line)
                throws InvalidIDException
```

:   Adds a line to the requested Category/Record.
    If the Category and Record already exist, the line is added to the
    end of the Record's list of lines. If the Category and/or Record
    does not exist, they will be created as needed and then the line
    will be added. If line is null, the Record will not add anything.

    :   **Parameters:**: `categoryName` - the name of the Category.: `recordName` - the name of the Record.: `line` - the line to add to the Record. **Throws:**: `InvalidIDException` - if categoryName or recordName is null or an empty String.

---


### getRecord

```
public Record getRecord(String categoryName,
                        String recordName)
```

:   Retrieves a Record by its Category name and Record name.
    Returns null if the requested Record does not exist.

    :   **Parameters:**: `categoryName` - the name of the Record's Category.: `recordName` - the name of the Record. **Returns:**: The requested Record.

---


### addNewCategory

```
public Category addNewCategory(String categoryName)
                        throws InvalidIDException
```

:   Creates a Category, adds it to a hash, and then returns the new
    Category.

    :   **Parameters:**: `categoryName` - the name of the Category. **Throws:**: `InvalidIDException` - if categoryName or recordName is null or an empty String.

---


### getCategory

```
public Category getCategory(String categoryName)
```

:   Retrieves a Category by its name.
    Returns null if the Category is not found.

    :   **Parameters:**: `categoryName` - the name of the Category. **Returns:**: The requested Category.

---


### iteratorCategories

```
public Iterator<Category> iteratorCategories()
```

:   Returns an Iterator for the Category objects held by this
    Description.
    The iteration order will be the same as the order in which the
    Category objects were added to this Description. If a Category
    is overriden with another Record of the same name, the replacement
    would not change the iteration order in any way.

    :   **Returns:**: An Iterator for the Category objects.

---


### numberOfCategories

```
public int numberOfCategories()
```

:   Returns the number of Category objects held by this Description.

    :   **Returns:**: The total number of Category objects.

---


### removeCategory

```
public void removeCategory(String categoryName)
```

:   Removes a Category.
    No action is taken if the Category does not exist.

    :   **Parameters:**: `categoryName` - the name of the Category.


---


|  |  |  |
| --- | --- | --- |
| **Overview** \| **Package** \| **Class** \| **Use** \| **Tree** \| **Deprecated** \| **Index** \| **Help** | |  |
| **PREV CLASS**   **NEXT CLASS** | **FRAMES**    **NO FRAMES**     **All Classes** |  |
| SUMMARY: NESTED \| FIELD \| CONSTR \| METHOD | DETAIL: FIELD \| CONSTR \| METHOD |  |


---

# *Copyright © 2007-2008*
